# The human exposome unraveling the impact of environment on health: promise or reality?

**DOI:** 10.11606/S1518-8787.2019053000649

**Published:** 2019-01-18

**Authors:** Kelly Polido Kaneshiro Olympio, Fernanda Junqueira Salles, Ana Paula Sacone da Silva Ferreira, Elizeu Chiodi Pereira, Allan Santos de Oliveira, Isabelle Nogueira Leroux, Flávia Bosquê Alves Vieira

**Affiliations:** I Universidade de São Paulo. Faculdade de Saúde Pública. Departamento de Saúde Ambiental. São Paulo, SP, Brasil; II Universidade de São Paulo. Faculdade de Saúde Pública. Departamento de Saúde Ambiental. Grupo de Pesquisa eXsat. Expossoma e Saúde do Trabalhador. The Human Exposome Research Group. São Paulo, SP, Brasil

**Keywords:** Environmental Pollutants, adverse effects, Risk Assessment, Epidemiology, Toxicology, Environmental Health, Exposure Science, Poluentes Ambientais, efeitos adversos, Medição de Risco, Epidemiologia, Toxicologia, Saúde Ambiental, Ciência da Exposição

## Abstract

Considering the innovative nature of the approach to human exposome, we present the state of the art of studies on exposome, and discuss current challenges and perspectives in this area. Several reading and discussion activities were conducted by the Expossoma e Saúde do Trabalhador (eXsat – Group Exposome and Worker's Health), with systematization of the literature in the area published between January 2005 and January 2017, available in the databases PubMed and Web of Science. This comment brings a thematic analysis to encourage the dissemination of the exposome approach for studies in the Public Health area.

## RESUMO

Levando em consideração a natureza inovadora da abordagem do expossoma humano, apresentamos o estado da arte dos estudos sobre expossoma, e discutimos os desafios e perspectivas atuais nessa área. Foram realizadas diversas atividades de leitura e discussão pelo grupo eXsat (Expossoma e Saúde do Trabalhador), com sistematização da literatura da área publicada entre janeiro de 2005 e janeiro de 2017, disponíveis nas bases de dados PubMed e Web of Science. O presente comentário traz uma análise da temática de forma a incentivar a disseminação da abordagem do expossoma nos estudos da área de Saúde Pública.

## INTRODUCTION

The progress in the study of exposure science began with the interest in the environmental origins of human diseases, which brought advances relevant to public and occupational health^1^ ^,^ ^2^. In the first half of the 20^th^ century, scientists and engineers from the health area already had instruments to measure the exposure available, which allowed a more enhanced building of the exposure-response relations for occupational diseases. As described by Rappaport^1^ (2011), the scientific studies on this subject were developed first in mines and factories and aimed at measuring the dust and chemical compounds transported by air. Between the 1950s and 1970s, the studies of urban pollutants began, including contaminants of water and air. While the first personal sampling techniques were carried out in workplaces in the 1960s, in the 1970s, the first American laws associated with exposure were created. These laws established the OSHA (Occupational Safety and Health Administration) to secure and oblige the compliance of the exposure limits in the workplace, and the EPA (Environmental Protection Agency) to assess risks and regulate contaminants in water and air. Despite the immeasurable progress that the creation of these agencies represented to the exposure science, the creation of these two agencies resulted in a separation between the professionals of the exposure area and divided the professional training and study design according to the exposure source, whether occupational or environmental^1^.

Considering this brief history, despite advances, the environmental and occupational toxicology began to be treated as independent areas with regard to the direction of projects and professional and scientific training. This separation encouraged research involving chemicals of regulatory importance primarily, as it discouraged the discovery of other sources of exposures that may be responsible for most diseases^1^. Several researches in environmental epidemiology deal with secondary data and concentrate investigations in particular exhibitions that evaluate the effects of the contamination of water, air, diet, stress, different life styles or types of infection on health outcomes. This information reflects the historical way of doing science, as a pie, in which each group of researchers deals with a piece. This limited division of the science pie leads to the scientific separation and confuses the definition of environmental exposures.

The modern toxicology investigates a wide range of dangers, both the old ones that still require confronting, as the emerging ones^3^. To reach their goals and advance in knowledge, the area has been supporting itself on tools related to genome and toxicogenomics. The great complexity of metabolic pathways, considering the exposure biology, must be studied when the interactions between genes and environment are investigated. During the life cycle, individuals are simultaneously exposed to a wide variety of factors. All categories of these exposure factors (general external exposure, specific external exposure and internal exposure) can contribute to the establishment of chronic diseases; thus, all the risk factors of interest should have been investigated collectively, and not individually^4^.

To better conduct the studies in this era of complexity of realities and avalanche of information, toxicology requires a closer connection with other disciplines, such as epidemiology and bioinformatics. We must consider that old dangers remain in the priority list to be studied and discussed, such as exposure to lead; however, daily, molecules are produced to satisfy the comfort of modern society. These molecules also have a biological effect. Between the discovery of a molecule and the study of its toxicity, there is a gap of time. In this period, the population may present health outcomes before the toxicity of the molecule is proven and its regulation, held.

The comprehension of the extent of genomic diversity among human beings, the recognition of the connection between phenotypes and diseases, and the discovery of environmental exposures that are harmful to our health are issues that continue to be faced by science^5^. In this sense, the fragmentation of epidemiological research symbolizes an obstacle in the identification of the main exposures^4^. The use of inappropriate risk models and the current limitation in measurement instruments can collaborate for the main environmental risk factors to remain unknown, underestimated or limited, as the exposures that impact on health the most may be in synergy with other exposures or biological or behavioral factors^6^. For this reason, studies based on molecular epidemiology are translated into an important tool to investigate the health effects in various exposure circumstances in humans. Biospecimen banks are of utmost importance to the studies on specific biological markers of certain diseases. In the field of toxicogenomics, the enzymes, their coding genes, and the metabolism of exogenous agents are used to investigate environmental factors from exposure to effect. The identification of the polymorphism of these genes indicates potential modifiers of the pathogenesis of environmental diseases^3^.

### Concept and Method of Exposome

In this context, the need of considering different exposures in the same epidemiological design led to the emergence of the concept of exposome. This term was coined by Wild^7^ in 2005 and comprises the totality of human exposures throughout life, from the conception to the death. Miller and Jones^8^ refined the concept of exposome as the cumulative measure of environmental influences and associated biological responses, including exposures of environment, diet, behavior and endogenous processes throughout life. The exposome is concomitantly based on three domains. Internal factors are those unique of individuals, such as physiology, age, body morphology and individual's genome; general external factors include socioeconomic condition, sociodemographic factors and place of residence; and specific external factors include diet, lifestyle, environmental and occupational exposures, among others^1^ ^,^ ^4^.

A coherent view of the environmental exposure acknowledges that the toxic effects are mediated by chemicals that alter critical molecules, cells and physiological processes within the body. Therefore, we can consider the environment as the internal chemical environment of the body and the exposures as the quantities of biologically-active chemicals in this internal environment^4^. In this view, exposures can originate from chemicals (toxicants) from the air, the water or the food. However, they include also chemicals produced by inflammation, oxidative stress, lipid peroxidation, inflammation, intestinal flora and other natural processes^4^ ^,^ ^9^ ^,^ ^10^. This internal chemical environment is dynamic because of changes in internal and external sources, aging, infections, lifestyle, stress, psychological factors and preexistent diseases^10^ ^,^ ^11^.

Recent listings show that humans are exposed to several chemical stressors throughout life. For example, by the US Toxic Substances Control Act, the EPA compiled 84,000 chemicals for which there is a risk of exposure. A total of 3,600 toxicants were identified in the Toxic Exposome Database and other 13,000 in the Comparative Toxigenomics Database^12^. Rappaport et al.^13^ related the risk of chronic diseases with the concentration of 1,561 chemicals in the blood, diet derivatives, pollutants, drugs or endogenous agents. From these chemicals, only 300 have been assessed in clinical and epidemiological studies, which indicates the importance of expanding the research beyond the endogenous metabolism, including the activity of several active chemicals. The study of exposome includes analyses of small molecules, products of metabolism (endogenous exposures); non-chemical stressors, such as radiation and climate; and exposure to complex mixtures such as air and water pollution. Endogenous factors such as oxidative stress, interaction between exogenous agents and metabolism, DNA repair mechanisms and mutations must also be considered when studying the human exposome^14^ ^-^ ^16^.

To evaluate several exposures simultaneously can provide a more accurate analysis of the impact of the environment on human health^6^. For that, the exposome characterization can follow two strategies: bottom-up and top-down. In the first, chemicals from each external source of an individual exposure are selected and measured at each point in time. In the second, chemicals and their metabolites are evaluated according to the toxicant profile and classes that cause diseases: metals, reactive electrophiles, endocrine disruptors, modulators of immune responses and agents that connect to cell receptors^4^ ^,^ ^10^ ^,^ ^17^ ^,^ ^18^. Considering these strategies, Rappaport^1^ proposes the application of a top-down approach based on biomonitoring with blood sampling. In this case, because exposure sources and levels change over time, the exposome can be characterized according to analysis of blood samples obtained in critical stages of life. More recently, the research group of the same author has proposed this biomonitoring with total saliva collection^19^.

To sequence the exposome, data analysis methods of high performance can be used to discover relationships between exposure, genome and diseases of interest, especially chronic diseases of unknown causes. There is evidence that genetic variants explain a limited fraction – 10% to 30% – of the variability of risk of chronic diseases^1^ ^,^ ^4^, which indicates the expressive role of environmental exposures and of the interaction between environmental and genetic factors^20^. Considering allergic diseases, for example, genetic studies provide insights about the mechanisms involved in its occurrence. The same reasoning can be applied in other cases, as in type II diabetes or obesity. Various environmental and lifestyle risk factors (urbanization, air pollution, occupational exposure, viral infections, smoking, diet, etc.) have been associated with the development of asthma and allergic diseases, which shows the importance of considering the set of exposures in the etiology of diseases^6^.

To discuss and evaluate the concept, one must make the distinction between the exposomic methodology and the underlying phenomenon to be measured^11^. The exposome is a compilation of non-genetic exposures that influence human health. Its study is conducted by the exposomic methodology, which involves simultaneous measurement of a set of biomarkers^21^, possible by the advances occurring in laboratory sciences. Currently, the simultaneous interpretation of thousands of individual compounds, such as metabolites, proteins, lipids and transcripts, is a reality. High-performance technologies such as omics (metabolomics, proteomics, adutomics, transcriptomics, lipidomics, etc.), allied to the evaluation devices of exposure to pollutants, and the evaluation questionnaires of past exposure and lifestyle form the measurement of the three aforementioned dimensions of the exposome: internal, general external, and specific external. The use of these technologies in epidemiological and longitudinal designs with powerful processing of large databases can lead to conclusions faster than in studies of isolated groups^22^. Statistical models that enable the integration of information obtained will be able to enhance risk assessment studies, to contribute to the disease prevention, and to generate accurate and pesonalized diagnoses to the medicine^23^.

### What has been Developed in Studies of Exposome?

Some readers may consider the concept of exposome discouraging, especially because of the idea of measuring all exposures to which an individual has been subjected throughout life and predicting their impact on health. However, large cohort studies have already been conducted in the world considering the principles of the exposome. Such studies have been increasing the accuracy of estimated associations between exposures, effects and health conditions^24^. The National Institute of Environmental Health Sciences (NIEHS) has been financing the concept of exposome, supporting studies that have been defined as exposomic in nature^18^. The institute outlined the objective of transforming the exposure science and has been identifying the application of the concept of exposome in its studies as a strategy. It plans to advance in the characterization of environmental exposure assessment, both at individual and population levels, through measurement tools and technologies at multiscale. The institute founded the project Hercules (Health and Exposome Research Center: Understanding Lifetime Exposures) with headquarters at Emory University (Atlanta, USA) and collaboration of universities such as Berkeley and Harvard^6^ ^,^ ^18^.

In Europe, several projects that consider exposomic approaches are in progress. The EXPOsOMICS is a research project, led by the Imperial College (London); the HELIX (The Human Early-Life Exposome) is mainly allocated in the *Instituto de Salud Global de Barcelona*; and the HEALS (Health and Environment-wide Associations based on Large population Surveys) have joint coordination of researchers from France and Greece. These organizations are heavily financed by the European Union and composed by research institutes from several countries.

## FINAL CONSIDERATIONS

In conclusion, the exposome is an ongoing reality and no longer a promise. The joint use of tools of epidemiology, bioinformatics and toxicology can promote the advancement of knowledge of the causes of public health outcomes to which the genomic sequencing has not clarified completely. Research in the public health area must, increasingly, include such tools in their population cohort studies. Succeeding in the characterization of the exposome, genetic and environmental determinants will be able to be concomitantly evaluated in the same study, in which interactions between gene and environment are examined^6^ ^,^ ^18^. Thus, the exposome represents a paradigm shift in the concept of making science, leaving the binomial unique exposure-outcome for the definitive recognition that health is impacted by multiple exposures. With the exposomic methodology available, such recognition can be achieved in new study designs^12^. This proposal will be able to solve the old impasse of what is nature (legacy) and of what is nurture (acquired)^4^.

We must consider that the concept of exposome is in full adolescence, with 13 years of age, since Christopher Wild coined it in 2005. Therefore, for the advancement of exposomic research, which integrate multiple scientific areas, there are the challenges of obtaining considerable financing; of the integration of knowledge, which determines the work of large research groups; and of the analysis of large databases, cross-sectional subject to several areas of knowledge, such as statistics, computer science, biomedicine, epidemiology and public health^3^ ^,^ ^12^ ^,^ ^25^. The difficulties in the field of bioinformatics are many because variables can be highly correlated and there are always risks of spurious correlations^12^. It is not enough to obtain data and perform correlations. The data must become reliable information and the epidemiology has key role in this point. Despite all the challenges discussed, the research on exposome is an ongoing reality and must include the training of a new body of scientists, who must be conscious of the need of a transdisciplinary education^12^. Such leaders in the area of human exposome must serve as a bridge between the multiple fields of research and work in partnerships or teams with scientific capabilities that complement each other and, consequently, advance in the answers that science seeks. With that, a new generation of researchers is expected, especially biomedical ones, epidemiologists, exposure and computing scientists, in addition to innovative and transcontinental research programs. The exposome opens a new era for thinking and working the science.
